# Are Chokeberry Products Safe for Health? Evaluation of the Content of Contaminants and Health Risk

**DOI:** 10.3390/foods12173271

**Published:** 2023-08-31

**Authors:** Ewa Olechno, Anna Puścion-Jakubik, Jolanta Soroczyńska, Katarzyna Socha, Małgorzata Elżbieta Zujko

**Affiliations:** 1 Department of Food Biotechnology, Faculty of Health Science, Medical University of Białystok, Szpitalna 37 Street, 15-295 Białystok, Poland; ewa.olechno@sd.umb.edu.pl; 2 Department of Bromatology, Faculty of Pharmacy with the Division of Laboratory Medicine, Medical University of Białystok, Mickiewicza 2D Street, 15-222 Białystok, Poland; jolanta.soroczynska@umb.edu.pl (J.S.); katarzyna.socha@umb.edu.pl (K.S.)

**Keywords:** toxic elements, chokeberry, nitrates and nitrites, conventional products, organic products, juices, fibers, environmental pollution, food safety

## Abstract

The health-promoting properties of chokeberry fruit have been confirmed in numerous scientific studies. It has been shown that the consumption of these fruits, due to the high content of bioactive compounds, has beneficial effects in neurodegenerative diseases, in addition to having hypolipemic, hypotensive, hypoglycemic, and anti-inflammatory properties. However, different conditions and methods of fruit cultivation, as well as methods of juice and fiber production, may result in a high content of toxic substances, which reduce the health value of chokeberry products. Many substances are environmental pollutants. In this study, for the first time, we examined the content of toxic elements (As, Hg, Cd, Pb), nitrates, and nitrites in all chokeberry juices (organic, conventional, from concentrate, and not from fruit concentrate) without additives and in all chokeberry fibers available in Poland. In addition, risk indicators of adverse health effects were calculated. The median content of the contaminants tested in juices was 0.461 µg/kg for As, 1.170 µg/kg for Cd, 0.427 µg/kg for Hg, 1.404 µg/kg for Pb, 4.892 mg/kg for NO_2_^−^, and 41.788 mg/kg for NO_3_^−^. These values did not exceed the permissible standards for the calculated indicators. There were also no statistically significant differences in the content of Cd, Hg, and Pb, as well as nitrates (III) and nitrates (V), in the tested juices depending on the method of cultivation and juice production. However, statistically significant differences in As content were found between juices from conventional and organic cultivation (1.032 µg/kg vs. 0.458 µg/kg) and juices from concentrate and not from concentrate (1.164 µg/kg vs. 0.460 µg/kg). There were no statistically significant differences with respect to impurities in fibers. It is shown that the consumption of chokeberry juice and fiber in the amount normally consumed does not pose a health risk associated with the intake of toxic substances; in the case of long-term fiber consumption, the Pb content should be monitored. In particular, organic juices and those not from fruit concentrate are recommended due to the lower As content.

## 1. Introduction

Currently, there is a growing interest in a healthy lifestyle, an integral part of which is a properly composed diet containing foods and juices with proven health-promoting effects, including prophylactic products. This food category includes fruits and juices made from 100% fruit without any additives. A special group of fruits is represented by chokeberry fruits, which have been the subject of scientific research for many years [1].

Aronia is a rich source of bioactive substances. It has been shown that regular consumption of chokeberry juice may have a hypolipemic, hypotensive, and hypoglycemic effect [2,3,4]. Aronia berries are a rich source of polyphenols (anthocyanins, procyanidins, phenolic acids, flavonols, and flavanols), vitamin C, macroelements (magnesium and potassium), and microelements (copper, iodine, iron, manganese, zinc, and selenium), as well as fiber [5]. The concentration of individual bioactive ingredients depends on the variety and ripeness of the fruit, agrotechnical and climatic conditions, technological processes, the form of administration, and the method of storing fruit or products [6,7,8].

Despite the high content of bioactive ingredients, chokeberry products can be contaminated, which is closely related to environmental pollution. Among the basic contaminants of natural products, the most important are toxic elements—lead (Pb), mercury (Hg), arsenic (As), and cadmium (Cd), nitrates, and nitrites [9,10,11]. Long-term exposure to environmental pollutants, including heavy metals, may result in an increased risk of metabolic disorders or neurological diseases, and may also cause genotoxicity. It is well known that toxic elements contribute to DNA damage and mutation, which in turn leads to carcinogenesis. This has been confirmed by the International Agency for Research on Cancer (IARC) [12,13].

The main source of human exposure to Hg is intake of contaminated fish and seafood, as well as inhalation of Hg vapors generated in industrial processes. Hg has a detrimental effect primarily on the central and peripheral nervous system, as well as the digestive system, immune system, lungs, kidneys, skin and eyes [14,15].

As exposure may be associated with consumption of drinking water and food, including some grains, fish, and seafood, and occupational exposure [16].

Pb naturally occurs in the Earth’s crust, but its widespread presence in the environment today is due to human activity. Pb exposure can occur through skin contact, inhalation, and consumption of contaminated water or food. Chronic exposure to this element has a negative effect on the bones, liver, kidneys, and endocrine, nervous, immune, and digestive systems [17].

The common sources of exposure to Cd are cigarette smoke, industry, and contaminated food. Chronic exposure to Cd can lead to kidney damage and vitamin D metabolism disorders, which in turn can lead to osteoporosis and osteomalacia [18].

High concentrations of nitrates (III) and (V) in food may result from excessive use of mineral and organic fertilizers, industrial pollution, or deliberate addition to food during processing. About 80% of nitrates in the diet come from the consumption of vegetables, mainly leafy ones, but also fruit and processed meat. Nitrates (V) in themselves are not harmful to humans, but their conversion to nitrates (III) by bacteria in the digestive tract is probably harmful [19,20]. Nitrates (III) can react with dietary amines to form carcinogenic nitrosamines [21]. There is still a lingering concern about nitrates in relation to methemoglobinemia [22]. On the other hand, clinical trials with dietary nitrate have shown a beneficial effect on cardiovascular diseases with a concomitant augmentation of markers of NO status [23].

The health-promoting properties of chokeberry fruit are well documented. However, there is a lack of studies about contaminants in chokeberry products, with only one from Serbia [1] and one from Croatia [24]. The authors assessed the content of toxic elements [1,24]. In addition, the content of mycotoxin patulin and hydroxymethylfurfural was assessed in the study by Torović et al. (2023) [1]. The authors emphasized that the consumption of chokeberry fruit, chokeberry juices, and chokeberry infusions does not pose a threat to human health [1,24]. However, it is worth emphasizing that no specified legal standards regarding the content of these substances in chokeberry fruit and chokeberry products have been developed so far.

In this study, for the first time, we examined the content of toxic elements (As, Cd, Hg, Pb), nitrates, and nitrites in all chokeberry juices (organic, conventional, from concentrate, and not from fruit concentrate) without additives and in all chokeberry fibers available in Poland. We would like to note that the range of tested products was very wide and included all available chokeberry juices and fibers at the time of collecting samples for research. In addition, risk indicators of adverse health effects were calculated to assess carcinogenic and non-carcinogenic risks.

## 2. Materials and Methods

### 2.1. Materials

The research material consisted of 25 100% chokeberry juices without additives. All juices were pasteurized. A total of 15 juices were organic juices, while 10 juices were conventional juices. Among these juices, 20 juices were not from fruit concentrate (NFC), and 5 juices were from fruit concentrate (FC). Additionally, six chokeberry fibers available on the market were also tested—three organic and three conventional. As part of the research work, most of the assortment available for sale in Poland was tested.

### 2.2. Methods

The contents of toxic elements (As, Cd, Hg, Pb) and nitrates (III) and (V) were determined in chokeberry juices and fibers.

Ultrapure water was used for all chemical determinations and was prepared with Simplicity 185 (Millipore, Burlington, NJ, USA). Standard solutions of toxic elements (As, Cd, Hg, Pb) were prepared on the basis of stock solutions of 1000 mg/L (Merck, Darmstadt, Germany). The samples were weighed with an accuracy of 1 mg. Spectrally concentrated ultrapure nitric acid 69% (4.0 mL, 69% HNO_3,_ Tracepur, Merck, Darmstadt, Germany) was used in the digestion step during the mineralization process. The mineralization process was carried out in a closed-loop microwave system (Speedwave, Berghof, Eningen, Germany). The volumes of samples after mineralization were noted; they ranged from 5.0 to 6.0 mL [25].

#### 2.2.1. Determination of As, Cd, and Pb Content in Chokeberry Juices and Fibers

As content was determined by inductively coupled plasma mass spectrometry (ICP-MS) (NexION 300 D ICP-MS, PerkinElmer, Boston, MA, USA) with a kinetic energy discrimination (KED) chamber, while the content of Cd and Pb was determined in the standard mode of ICP-MS. The limits of detection for As, Cd, and Pb were 0.019 µg/kg, 0.017 µg/kg, and 0.016 µg/kg. All determinations were performed in 5 replicates.

During estimation toxic elements by ICP-MS method, the following conditions were preserved:

(a) For As: mass—75 amu, dwell time per amu (ms)—50, integration time (ms)—1000, detector calibration mode—dual,
(b) For Cd: mass—110, 111, 113 and 114 amu, Dwell time per amu (ms)—50, integration time (ms)—1000, detector calibration mode—dual,
(c) For Pb: mass—206, 207 and 208 amu, Dwell time per amu (ms)—50, integration time (ms)—1000, detector calibration mode—dual.

#### 2.2.2. Determination of Hg Content in Chokeberry Juices and Fibers

The determination of the Hg content in the tested samples was carried out using the atomic absorption spectrometry (ASA) method by AMA-254 (Advanced Mercury Analizer 254, Leco, Altec, Praha, Czech Republic).

The samples were properly weighed, then put in a cuvette and analyzed. Next, samples were dried and burned in oxygen (600 °C). The Hg vapor was then trapped by the gold amalgamator. The released Hg was then measured under the following conditions: drying time—60 or 70 s; decomposition time—150 or 120 s; waiting time—45 or 40 s. The detection limit for Hg was 0.005 ng/L. Results were presented in µg per kg of juice and in µg per kg of fiber.

#### 2.2.3. Determination of Nitrates and Nitrites Content in Chokeberry Juices and Fibers

Nitrates (III) and (V) were determined by the spectrophotometric method using Griess I and II reagents (all reagents were purchased from Sigma Aldrich, Saint Louis, MI, USA).

The sample (5 mL of the chokeberry juice and 5 g of the chokeberry fiber) was transferred with 100 mL of water at a temperature of 70–80 °C to a 200 mL volumetric flask. Then, hydrated borate of sodium was added (5 mL) and shaken vigorously several times. The flask was heated in a shaker water bath for 30 min at 90–100 °C. After 30 min, the sample was cooled, and 2 mL of potassium hexocyanoferrate (II) and 2 mL of zinc acetate (II) were added successively. The samples were shaken after each addition of reagents. Then, the samples were filled to the mark with water (200 mL), mixed, and filtered through filter white. Next, 10 mL of the filtrate was taken for determinations. Activated carbon was used to obtain a colorless solution.

Firstly, 10, 20, or 30 mL of the filtrate were transferred into a 50 mL volumetric flask, depending on the expected amount of nitrates (III) and diluted with distilled water to 30 mL if necessary. Griess I reagent (5 mL) was added and left in the dark for 5 min. Then, Griess II (1 mL) reagent was added, mixed, and left out of the light for 10 min. The sample was filled with water to the mark, and, after 20 min, the absorbance (wavelength: 538 nm) against the standard was measured.

In the next stage, nitrates (V) were reduced to nitrates (III). For this purpose, 10 mL of the filtrate was pipetted into a 50 mL flask to which 5 mL of ammonium buffer and 2 g of Cd were added. The solution was shaken for 30 min. The contents of the flask were then filtered through filter paper. Then, the analytical cycle was repeated as in the case of determining the content of nitrates (III).

The content of nitrates (III) and (V) was calculated using the appropriate formulas.

Nitrate (III) content (X) expressed as nitrate (III) ion (NO_2_^−^) in mg/kg of product calculate according to the following formula:

$$X=m_{1}\times 200\frac{200}{V_{1}\times m_{0}}$$

where m_1_ is the mass of ions (NO_2_^−^) contained in V_1_ volume of the filtrate read from the graph calibration (μg), V_1_ is the volume of filtrate taken for spectrophotometric determination (mL) m_0_ is the mass of sample taken for determination (g), and 200 is the total filtrate volume (mL).

The nitrate (X_2_) content expressed as nitrate (V) ions (NO_3_^−^) in mg/kg of product was calculated according to the following formula:

X_2_ = 1.348 × [(m_2_ × 10,000/V_2_ × V_3_ × m_0_) − X)]

where m_0_ is the sample mass taken for determination (g), m_2_ is the total mass of nitrate (III) as ion (NO_2_^−^) contained in V_3_ volume of the filtrate and read from the calibration graph (µg), V_2_ is the volume of the filtrate taken for reduction of nitrate (V) (mL), V_3_ is the volume of the filtrate taken for photometric determination (mL), X is the nitrate (III) content determined in the sample (mg/kg), 10,000 is the the product of 200 × 50, where 200 is the total volume of the filtrate (mL) and 50 is volume of solution after reduction of nitrate (V) (mL), and 1.348 is the ratio of the molecular weight of the ion (NO_3_^−^) to the ion (NO_2_^−^). The calibration graph was drawn up to the value of 30 μg of ions (NO_2_^−^) [26].

### 2.3. Estimation of Human Health Risk

The risk of adverse health effects resulting from the intake of the studied chemicals from chokeberry product consumption was assessed by calculating for each element selected indicators: estimated daily intake (EDI), provisional tolerable weekly intake (PTWI), tolerable weekly intake (TWI), benchmark dose lower confidence limit (BDML), carcinogenic risk (CR), target hazard quotient (THQ), and hazard index (HI). As recommended by the Joint FAO/WHO Expert Committee on Food Additives (JECFA), different indicators were used for the studied elements. For Hg, PTWI was estimated, and, for Cd, TWI was estimated. For As and Pb, the BDML_0.5_ (the benchmark dose lower confidence limit for a 0.5% increased incidence of lung cancer) was identified. We determined these indicators in order to evaluate the possible risk of short- and long-term adverse health effects.

The results were recalculated for an adult weighing 70 kg, using a standard portion of studied products. We estimated the portion of chokeberry juice as 100 mL per day and portion of chokeberry fiber as 10 g per day. These amounts were suggested by the manufacturers of chokeberry juices and fibers.

The calculations were based on EFSA regulations. Appropriately, PTWI for Hg was 4 µg/kg/week, TWI for Cd was 2.5 µg/kg/week, BMDL for Pb was 0.02–3 µg/kg/day, and BMDL for As was 3 µg/kg/day [27,28,29]. BMDL values determine the lowest doses associated with the development of a specific effect on the human body. We compared our results with the maximum BMDL values of these toxic elements for a person weighing 70 kg.

The EDI (mg/kg bw/day) evaluates the daily intake of a specific element from food. It is a common index calculated to determine the transfer of investigated elements to the human body via food consumption. So far, there have been no regulations evaluating EDI for the consumption of aronia products. The EDI for the studied elements was assessed by multiplying the mean content of an element determined in the chokeberry juice and fiber samples by the daily portion of these products consumed by a person with an average body weight of 70 kg, using the following formula:

EDI = FIR × C/BW,

where FIR is the estimated consumption of chokeberry juice or chokeberry fiber per day (as 100 mL per day for chokeberry juice and 10 g per day for chokeberry fiber) in accordance with the manufacturer’s recommendations, and, in the case of chokeberry juice, additionally in accordance with literature data [4], C is the mean element concentration in chokeberry juices and fibers samples (mg/kg), and BW is the body weight (70 kg).

PTWI = EDI × 7

The PTWI value for Hg was assumed, in line with JECFA guidelines, as 4 µg/kg BW/week [30].

The CR indicator is used to evaluate value is a measure of the probability of a cancer risk developing during a lifetime as a result of exposure to toxic substances, including toxic elements a consequence of exposure to carcinogens, such as Pb, Cd, and As [31]. If the value of CR exceeds 10^−4^, it means an increased high risk of developing cancer [32,33]. A CR value less than 10^−4^ indicates acceptable risk. CR was calculated using the following formula:

CR = Efr × EDtot × EDI × CSf/ATn,

where Efr is the exposure frequency (365 days per year), EDtot is the exposure duration (70 years), EDI is the daily intake (mg/kg/day), and ATn is the average exposure in a year (365 days/year × 70 years). CSf is 1.5 for As, 6.3 for Cd, and 0.0085 mg/kg per day for Pb [34].

The THQ indicator was used to estimate long-term non-carcinogenic health risk associated with intake of studied toxic elements (As, Cd, Hg, Pb) from chokeberry juice and chokeberry fiber. If the value of the THQ is >1, it means that there is some health risk associated with the consumption of chokeberry products. The THQ was assessed using the following formula:

THQ = (Efr × EDtot × EDI)/(RfDo × ATn),

where Efr is the exposure frequency (365 days per year), Edtot is the exposure duration (70 years), EDI is the estimated daily intake (mg/kg bw/day), RfDo is the oral reference dose specific for every element (As—0.0003 mg/kg/day, Cd—0.001 mg/kg/day, Hg—0.0003 mg/kg/day, Pb—0.0035 mg/kg/day), BW is the body weight (70 kg), and ATn is the average exposure in a year (365 days/year × 70 years) [34].

The HI index was calculated as a sum of the THQs of all the negative health effects of the studied elements (As, Cd, Hg, and Pb) found in the samples of chokeberry products:

$$\mathrm{HI}=\sum_{i=k}^{n}{THQ}_{s},$$

where THQs is the target hazard quotient estimated for specific element. If HI is higher than 1, it is considered a significant health risk [35,36].

### 2.4. Statistical Analyses

Statistical analyses were created using the Statistica v.13.3 software (StatSoft, Krakow, Poland). We used the Shapiro–Wilk test, Lilliefors test, and Kolmogorov–Smirnov test to define the normality of the data. The assessment of statistical differences was carried out using the Mann–Whitney U test (*p*-values <0.05 denote statistical significance). Correlations of the results were determined using Spearman’s rank correlation coefficient.

## 3. Results

The results obtained are presented in Table 1, Table 2, Table 3 and Table 4. Table 1 shows the content of studied toxic elements, nitrates, and nitrites in chokeberry juices. Table 2 presents the content of studied contaminants in chokeberry fibers. Table 3 and Table 4 present the estimated health risk in relation to the content of toxic elements using appropriate indicators. 

Table 1 presents the content of the tested impurities in the juices. The median As content in the tested juices was 0.461 µg/kg. Statistically significant differences in As content were found between juices from conventional and organic cultivation (1.032 µg/kg vs. 0.458 µg/kg, respectively) and juices from concentrate and not from concentrate (1.164 µg/kg vs. 0.460 µg/kg). The median content of the remaining toxic metals tested (Cd, Hg, and Pb) was respectively 1.170 µg/kg, 0.427 µg/kg, and 1.404 µg/kg. The median content of nitrates (III) was 4.892 mg/kg, and that of nitrates (V) was 41.788 mg/kg. There were no statistically significant differences in the content of Cd, Hg, and Pb, as well as nitrates (III) and nitrates (V), in the tested juices depending on the method of cultivation and juice production.

Table 2 presents the content of the tested environmental pollutants in chokeberry fibers. The type of cultivation had no effect on the content of pollutants. It is worth emphasizing, however, that the Pb content was about three times higher in conventional fibers than in organic fibers (65.133 vs. 21.273 µg/kg).

The health risk indicators are shown in Table 3 and Table 4. In the case of BMDL and PTWI, the limits were not exceeded (Table 3). Table 3 shows the values of the BMDL indicators. The obtained values for As and Pb were definitely lower than the reference limit in the case of both chokeberry juices and fibers. The level of TWI and PTWI of Hg and Cd in chokeberry juices and fibers was also low (Table 3). The volume of juice (100 mL) covered 0.477–0.564% TWI for Cd and 0.137–0.142% PTWI for Hg. The volume of juice that would need to be consumed to cover the TWI for Cd is 20.83 L/week for conventional juices and 16.67 L/week for organic juices. When it comes to fiber, to cover the TWI of Cd, 1.45–2.78 kg of chokeberry fiber should be consumed per week. To cover the PTWI of Hg, an average of 71.43 L of conventional chokeberry juice, 71.43 L of organic chokeberry juice, and 6.67–11.1 kg of chokeberry fiber should be consumed per week. On the basis of the results obtained, EDI values ranged from 7.81 × 10^−7^ mg/day for Hg (conventional juices) to 2.69 × 10^−6^ mg/day for Pb (conventional juices). For chokeberry fiber, the EDI values ranged from 5.35 × 10^−7^ mg/day for Hg (organic fibers) to 9.31 × 10^−6^ mg/day for Pb (conventional fibers) (Table 4). The highest percentage of BMDL was found for Pb in the case of conventional chokeberry products: from 0.310% to 4.656% for fibers and from 0.090% to 1.344% for juices. The values of BMDL for As were also higher for conventional products. The amount of conventional fiber covered 0.170% of the BMDL, and the volume of conventional juice covered 0.047% of the BMDL.

The assessed THQ, HI, and CR are summarized in Table 4. The THQ for all tested samples was below 1. Therefore, no increased risk was found due to the intake of the tested toxic elements. The THQ index was higher for Cd and Hg in the case of organic juices compared to traditional juices, while the THQ for Pb and As was lower for organic juices. Conventional chokeberry fibers had a higher THQ index for As, Hg, and Pb than organic fibers.

The CR value was below 10^−4^ for almost all products tested, with the exception of fibers for Pb. Our results, therefore, indicate that the risk of developing cancer from the consumption of the tested products is low.

## 4. Discussion

Table 5 shows the content of nitrates and nitrites in different types of juices. Table 6 presents the content of toxic elements in other products. Table 7 shows some of the health risk indicators calculated by other authors.

### 4.1. Arsenic Content

The median content of As in chokeberry juices was 0.461 µg/kg of juice. According to the latest Regulation of the European Commission from 2023, the maximum level of As for fruit juices is 0.02 mg/kg of fresh weight (20 µg/kg of fresh weight) [53]. None of the tested chokeberry juices exceeded this value. Both the method of cultivation and the restrictions on the use of fertilizers in the case of organic farming could have played a role. It has been shown that fertilizers may contain toxic elements [54,55]. In the study by Śmiechowska et al. (2011), the method of cultivation did not play a role in the content of toxic elements (in carrots, parsley, and potatoes), including the content of As [56]. Comparing the As content in chokeberry juices to other fruit juices, in the study by Dehelean et al. (2013), apple, peach, apricot, orange, kiwi, pear, and pineapple juices were characterized by a varied As content. Apple juice contained an undetectable value to about 4.36 µg/kg. Peach juice contained an undetectable value to about 3.78 µg/kg, while orange juice contained an undetectable value to about 3.02 µg/kg, and pineapple juice contained an undetectable value to about 2.84 µg/kg. In turn, As was not detected in kiwi and pear juices [42]. Unfortunately, there are no studies evaluating the content of toxic elements in other fibers available on the market.

### 4.2. Cadmium Content

The content of Cd in conventional chokeberry juices was 1.171 µg/kg, while that in organic juices was 1.067 µg/kg. The Regulation of the European Commission of 2021 set the maximum level of Cdfor berries at the level of 0.03 mg/kg of fresh weight (30 µg/kg of fresh weight) [57]. In the study by Gąstoł et al. (2012), it was noticed that organic juices (apple, pear, blackcurrant) contained surprisingly higher concentrations of Cd than conventional juices [47]. Similarly, in the study by Staniek et al. (2013), the evaluated organic products (cereal products, vegetables, fruit) contained higher concentrations of Cd than conventional products [58]. In another study, carrots from organic farming contained significantly lower levels of Cd than carrots from conventional farming [59]. Comparing the content of Cd with other fruit juices, the concentration of Cd was both similar, higher and lower. Aronia juices in the studies of other authors had a much higher content of Cdthan in our study: 16 µg/kg in the study by Krejpcio et al. (2005) and 14.8 µg/kg in the study by Torović et al. (2023) [1,41]. In turn, dried chokeberry fruits in the study Juranović Cindrić et al. (2017) contained 55 µg of Cd [24]. These differences may result from the origin of the fruit and the accumulation of elements in the soil. Conventional apple juice contained about 0.14 to 1.42 µg Cd/kg of juice in the study by Dehelean et al. (2013). In the study by Gąstoł et al. (2012), a kilogram of apple juice provided about 3 µg of Cd. Peach juice provided about 0.52 to 11.3 µg Cd/kg depending on the producer and origin of the juice [42,47]. In the study by Mohammed et al. (2020), no Cd was detected [51]. Orange juice contained 0 to about 20 µg/kg [42,45,50,51]. In the study by Gąstoł et al. (2012), blackcurrant juice provided higher amounts of Cd than chokeberry juice, about 7 µg/kg of juice [47].

### 4.3. Lead Content

The median content of Pb in conventional juices was 1.402 µg/kg, while, in organic juices, it was 1.503 µg/kg. The European Commission regulation from 2021 set the maximum allowable Pb content for berry juices at 50 µg/kg of fresh weight [60]. The juices assessed by us contained a much lower concentration of this element. In the Krejpcio et al. study (2005), traditional chokeberry juice contained significantly more Pbthan in our study—110 µg/kg of juice [41]. Torović et al. (2023) also showed slightly higher values for Pb in chokeberry juices, appropriately 7.6 µg/kg of juice [1]. Dried chokeberry fruits contained 41 µg/kg which may have resulted from water loss [24]. If we compare chokeberry juice with other fruit juices, it contained both lower and higher Pb content depending on the fruit. For example, the Pb content in apple juice in the study by Dehelean et al. (2013) was about 4.66–75.68 µg/kg depending on the juice brand, while the study by Williams et al. (2008) found about 80 µg/kg, and the study by Gąstoł et al. (2009) found about 9 µg/kg of juice [42,45,47]. Orange juice contained about 1.02 [42] to 80 µg/kg of juice [45]. Pear juice provided about 1.62 [42] to 10 µg Pb/kg, and blackcurrant juice contained about 15 µg/kg [47]. However, it should be emphasized that the diversified content of Pb in juices depends to a large extent on the origin.

### 4.4. Mercury Content

The median content of Hg in conventional juices in our study was 0.443 µg/kg, while that in organic juices was 0.427 µg/kg. According to the Regulation of the European Commission from 2018, the highest permissible level of Hg compounds in berries is 0.01 mg/kg (10 µg/kg of the product). The issued legal act does not include the maximum permissible amounts in the case of fruit juices or products such as fiber [48]. It seems, however, that the chokeberry products we tested contained significantly lower levels of Hg than the legal limits. In addition, it was noted that the concentration of nitrates (V) in juices was significantly correlated with the Hg content (*p* < 0.05). This may be due to the fact that Hg compounds may be used in the production of these fertilizers [61,62]. In addition, these fertilizers may increase the bioavailability of Hg and the relative amount of Hg-methylating microorganisms, but research is mainly concerned with the relationship of fertilizers used in rice fields [63,64]. There are no studies about Hg content in chokeberry juices and fibers by other researchers. There is also no research about the Hg content in similar products for other fruit or vegetable juices. In the study by Wojciechowska-Mazurek et al. (1995), the Hg content in selected fresh fruit in Poland was assessed. The median concentration of Hg was <1 to 2 µg/kg fresh weight for red currants, <1 to 1 µg/kg fresh weight for strawberries and cherries, and <1 µg/kg fresh weight for raspberries, blackcurrants, hungarian plums, apples, and pears [65]. Wyka et al. (2009) assessed the Hg content in various vegetables and fruits in one of the regions of Poland. Among fruits, the average content in apples was 0.13 µg/kg fresh weight, whereas, in pears, it was 0.1 µg/kg fresh weight [66]. The location of the crop could impact the Hg content.

### 4.5. Nitrates Content

The acceptable daily intake of nitrates (ADI) applies to food additives; therefore, it is impossible to relate the obtained results and estimate the risk of possible excess. It seems, however, that the obtained amounts of nitrates are small, taking into account the fact that the permissible maximum daily intake of nitrates for other plant products is much higher. For example, the maximum allowable level of nitrates for fresh lettuce is 2000 to 5000 mg/kg depending on the type of lettuce and time of harvest, while that for fresh spinach is 3500 mg/kg [67]. So far, the content of nitrates in chokeberry juices has not been determined; therefore, the comparison of their concentration is possible only in relation to other fruit and vegetable juices (Table 5). Chokeberry juice, from both conventional and organic cultivation, was characterized by a higher content of nitrates (V) than apple juice [37], orange juice [37,40], cherry juice, mango juice, and pineapple juice, but a similar content to grape juice [40]. On the other hand, compared to blackcurrant juice, it contained a similar or lower content of nitrates (V) [37]. Two blackcurrant juices in Śmiechowska et al.’s (2003) study contained about 54.6 and 81.9 mg of nitrates per kg of juice. Comparing chokeberry juices to vegetable juices, they contained a significantly lower content of nitrates (V) than beetroot juice [37,38]. Beetroot juice is known for its high content of nitrates, thanks to which it has a hypotensive effect [68]. The content of nitrates (V) in beetroot juice was about 938.9 mg/kg in the study by Śmiechowska et al. (2003) and about 1707–2625 mg/kg in the study by Tamme et al. (2011) [37,38]. Carrot juice is characterized by a varied content of nitrates (V), both higher and lower than juice from chokeberry: about 7.4 mg/kg for organic juice [37], in the range of about 32.9–419.5 for juice from conventional cultivation [37,38], and about 163 mg/kg for fresh juice [38]. Tomato juices contain a lower content of nitrates (V) (about 16.9–21.2 mg/kg), while fresh and cabbage juice contain a higher content of nitrates (V): about 116 [38] and 232.2 mg/kg for fresh juice [37], about 250 mg/kg for commercial juice [38], and about 94.6 mg/kg for organic cabbage juice [37].

In the case of nitrates (III), chokeberry juice was characterized by a lower content than pineapple, cherry, mango, and grape juice [40], and a higher content than apple juice, blackcurrant [37], carrot [37,38], tomato [37], cabbage [38], and beetroot [37]. In the case of orange juice, it did not contain [37] or contained a higher content of nitrates (III) (about 6.52 mg/kg) [39] compared to chokeberry juice.

The studies by Torović et al. (2023) and Juranović Cindrić et al. (2017) evaluated Cd and Pb [1,24] as well as boron, aluminum, cobalt, beryllium, nickel, and chromium [24]. Torović et al. (2023) assessed health risk using the hazard quotient (HQ) and HI index. HI for Cd and Pb was calculated at the level of 0.072–0.491 for eight chokeberry juices [1]. Our study showed a lower HI for both conventional and organic products. In turn, Juranovic Cindrić et al. (2017) estimated the PTWI for dried aronia fruits. According to the authors, consumption of 100 g of dried chokeberry would provide 0.069 µg of Cd and 0.051 of Pb per week [24].

In the research of other authors, the health risk for selected plant products was not shown either (Table 6) [43,44,49,52].

To sum up, there were no exceedances in the content of impurities; therefore, the values of the indicators presented in Table 3 and Table 4 confirm that health-promoting juices and fibers are safe and can be used prophylactically in various groups of patients. The exception is the CR index for fibers in the case of Pb; attention should be paid to this toxic element in case of long-term consumption.

## 5. Conclusions

Chokeberry products provide low amounts of toxic elements, nitrates, and nitrites. Their consumption does not pose a health risk in the amounts normally consumed by consumers. The type of cultivation had no significant effect on the content of impurities, except for arsenic. Chokeberry juices and fibers can be recommended as a supplement to the diet with beneficial bioactive substances, featuring a low content of pollutants.

## Figures and Tables

**Table 1:** Content of contaminants in juices.

	Average Content of Analyzed Parameters						
Type of Chokeberry Juices		As (µg/kg)	Cd (µg/kg)	Hg (µg/kg)	Pb (µg/kg)	NO_2_^−^ (mg/kg)	NO_3_^−^ (mg/kg)
Conventional (*n* = 10)	Med.	1.032 *	1.171	0.443	1.402	4.672	43.872
	Q1–Q3	0.461–1.313	1.106–1.198	0.241–0.961	1.123–1.623	4.584–5.204	38.143–49.310
	Av. ± SD	0.977 ± 0.428	1.194 ± 0.330	0.547 ± 0.347	1.882 ± 1.746	4.897 ± 0.586	43.927 ± 8.445
	Min–Max	0.405–1.525	0.567–1.928	0.186–1.048	0.830–6.787	4.272–6.132	32.956–60.461
Organic (*n* = 15)	Med.	0.458 *	1.067	0.427	1.503	4.892	41.788
	Q1–Q3	0.215–0.892	0.868–1.952	0.354–0.754	1.197–2.084	4.360–5.112	37.431–44.640
	Av. ± SD	0.556 ± 0.389	1.409 ± 0.732	0.568 ± 0.340	1.610 ± 0.793	4.824 ± 0.487	40.302 ± 5.533
	Min–Max	0.195–1.422	0.369–3.200	0.159–1.328	0.713–3.561	3.916–5.644	30.136–48.587
NFC (*n* = 20)	Med.	0.460 *	1.129	0.449	1.463	4.892	42.144
	Q1–Q3	0.294–0.929	0.908–1.862	0.361–0.989	1.143–1.953	4.448–5.136	38.175–45.845
	Av. ± SD	0.631 ± 0.448	1.376 ± 0.665	0.606 ± 0.351	1.810 ± 1.371	4.872 ± 0.557	41.288 ± 6.006
	Min–Max	0.195–1.525	0.369–3.200	0.159–1.328	0.713–6.787	3.916–6.132	30.136–50.507
FC (*n* = 5)	Med.	1.164 *	1.172	0.274	1.400	4.628	38.493
	Q1–Q3	0.901–1.218	1.170–1.172	0.241–0.517	1.227–1.404	4.584–5.112	37.431–47.099
	Av. ± SD	1.098 ± 0.191	1.113 ± 0.146	0.372 ± 0.186	1.355 ± 0.191	4.778 ± 0.363	43.608 ± 10.517
	Min–Max	0.892–1.313	0.853–1.198	0.204–0.623	1.123–1.623	4.360–5.204	34.557–60.461
Total (*n* = 25)	Med.	0.461	1.170	0.427	1.404	4.892	41.788
	Q1–Q3	0.405–1.164	0.908–1.680	0.303–0.754	1.197–1.633	4.536–5.112	38.143–46.619
	Av. ± SD	0.724 ± 0.448	1.323 ± 0.604	0.560 ± 0.336	1.719 ± 1.236	4.853 ± 0.518	41.752 ± 6.920
	Min–Max	0.195–1.525	0.369–3.200	0.159–1.328	0.7136.787	3.916–6.132	30.136–60.461

Av.—average, FC—from concentrate, Max—maximum value, Med—median, Min—minimum value, NFC—not from concentrate, Q1—lower quartile, Q3—upper quartile, SD—standard deviation. * Statistically significant difference (*p* < 0.05).

**Table 2:** Content of studied contaminants in chokeberry fibers.

Type of Chokeberry Fiber		As (µg/kg)	Cd (µg/kg)	Hg (µg/kg)	Pb (µg/kg)	NO_2_^−^ (mg/kg)	NO_3_^−^ (mg/kg)
Conventional Fiber (*n* = 3)	Med.	35.455	8.302	5.622	65.133	5.000	45.030
	Q1–Q3	30.212–41.251	7.101–11.344	5.333–6.578	60.222–70.185	4.520–5.732	30.907–48.003
	Min–Max	30.212–41.251	7.101–11.344	5.333–6.578	60.222–70.185	4.520–5.732	30.907–48.003
	Av. ± SD	35.639 ± 5.522	8.916 ± 2.187	5.844 ± 0.651	65.180 ± 4.981	5.084 ± 0.610	41.313 ± 9.134
Organic Fiber (*n* = 3)	Med.	29.899	17.183	3.742	21.273	5.424	42.524
	Q1–Q3	6.653–53.145	14.638–19.728	3.401–4.084	19.517–23.029	5.350–5.424	32.832–52.216
	Min–Max	6.653–53.145	14.638–19.728	3.401–4.084	19.517–23.029	5.350–5.424	32.832–52.216
	Av. ± SD	29.899 ± 23.246	17.183 ± 2.545	3.742 ± 0.341	21.273 ± 1.756	5.399 ± 0.043	42.524 ± 9.692
Total (*n* = 6)	Med.	32.834	12.991	4.708	41.626	5.387	43.777
	Q1–Q2	29.899–41.251	8.302–17.183	3.742–5.622	21.273–65.133	5.000–5.424	32.832–48.003
	Min–Max	6.653–53.145	7.101–19.728	3.401–6.578	19.517–70.185	4.520–5.732	30.907–52.216
	Av. ± SD	32.769 ± 15.435	13.049 ± 5.001	4.793 ± 1.242	43.226 ± 24.276	5.242 ± 0.424	41.919 ± 8.449

**Table 3:** BMDL, PTWI, and TWI values and content per serving in studied chokeberry products.

	*n*	As	Cd	Hg	Pb
Type of Product		%BMDL Per Serving #^,a^	%TWI Per Serving #^,a^	%PTWI Per Serving #^,a^	%BMDL Per Serving #^,a^
**Chokeberry Juices**					
Conventional	10	0.047	0.477	0.137	0.090–1.344
Organic	15	0.026	0.564	0.142	0.077–1.150
**Chokeberry Fibers**					
Conventional	3	0.170	0.357	0.146	0.310–4.656
Organic	3	0.142	0.687	0.094	0.101–1.520
Reference Limit		As: 3 µg/kg BW/day,	Cd: 2.5 µg/kg BW/week	Hg: 4 µg/kg BW/week	Pb: 0.2–3 µg/kg BW/day,
		210 µg/day ^a^	175 µg/week ^a^	280 µg/week ^a^	1.4–210 µg/day ^a^

BMDL—benchmark dose lower confidence limit, BW—body weight, PTWI—provisional tolerable weekly intake, TWI—tolerable weekly intake. ^a^ Amount for a person weighing 70 kg; # 100 mL or 10 g.

**Table 4:** The values of EDI, THQ, CR, and HI for studied chokeberry products.

	As			Cd			Hg			Pb			
Product	EDI	THQ	CR	EDI	THQ	CR	EDI	THQ	CR	EDI	THQ	CR	HI
**Juices**													
Conventional	1.40 × 10^−6^	4.65 × 10^−3^	2.09 × 10^−6^	1.71 × 10^−6^	1.71 × 10^−3^	1.07 × 10^−5^	7.81 × 10^−7^	2.60 × 10^−3^	NA	2.69 × 10^−6^	7.68 × 10^−4^	2.28 × 10^−8^	9.73 × 10^−3^
Organic	7.94 × 10^−7^	2.65 × 10^−3^	1.19 × 10^−6^	2.01 × 10^−6^	2.01 × 10^−3^	1.27 × 10^−5^	8.11 × 10^−7^	2.70 × 10 ^−3^	NA	2.30 × 10^−6^	6.57 × 10^−4^	1.96 × 10^−8^	8.02 × 10^−3^
**Fibers**													
Conventional	5.09 × 10^−6^	1.70 × 10^−2^	7.64 × 10^−6^	1.27 × 10^−6^	1.27 × 10^−3^	8.02 × 10^−6^	8.35 × 10^−7^	2.78 × 10^−3^	NA	9.31 × 10^−6^	2.66 × 10^−4^	1.70 × 10^−2^	2.13 × 10^−2^
Organic	4.27 × 10^−6^	1.42 × 10^−2^	6.41 × 10^−6^	2.45 × 10^−6^	2.45 × 10^−3^	1.55 × 10^−5^	5.35 × 10^−7^	1.78 × 10^−3^	NA	3.04 × 10^−6^	8.68 × 10^−5^	1.70 × 10^−2^	1.86 × 10^−2^

CR—carcinogenic risk, EDI—estimated daily intake, EWI—estimated weekly intake, HI—hazard index, NA—not applicable, THQ—target hazard quotient.

**Table 5:** Content of nitrates and nitrites in different type of juices determined by other authors.

Type of Juice	NO_2_^−^ (mg/kg)	NO_3_^−^ (mg/kg)	References
Apple	3.1–7.7	0.07–0.44	[37]
Beetroot	938.9	1.06	[37]
Beetroot	1707–2625	3.2	[38]
Blackcurrant	54.6–81.9	0.36–0.77	[37]
Cabbage	94.6–232.2	1.06	[37]
	116–250	0.6	[38]
Carrot	7.4–419.5	0.02–0.97	[37]
	87	0.1	[38]
Cucumber	42.7	9.03	[39]
Cherry	16.54	6.75	[40]
Grape	30.16	8.34	[40]
Mango	26.43	9.77	[40]
Melon	33.64	7.65	[39]
Orange	1.4	0	[38]
	19.56	6.52	[37]
Pineapple	24.25	6.83	[38]
Tomato	16.9–21.2	0.04–0.29	[37]
	7.82	1.81	[39]
Watermelon	26.61	5.5	[39]

**Table 6:** Content of As, Cd, Hg, and Pb in different type of products determined by other authors.

Type of Product	Type of Toxic Element			
	As	Cd	Hg	Pb
**Aronia Products**				
Aronia juice	-	16 µg/L [41]	-	110 µg/L [41]
Aronia juice (cold pressed)	-	14.8 µg/L [1]	-	7.6 µg/L [1]
Aronia berries (dried)	<LOD [24]	55 µg/kg DM [24]	-	41 µg/kg [24]
**Different Fruit Juices**				
Apple	<LOD—4.36 µg/L [42], 0.064 mg/kg [43], 1.6–1.8 µg/L [44]	<LOD [45,46], 0.4–0.5 µg/L [44], 3 µg/kg/FM (C,O) [47], 11 µg/kg [43], 16 µg/L [41], 240–1420 µg/L [48]	1 µg/L [44]	4.66–75.68 µg/L [42], 10 µg/kg/FM (O), 8 µg/kg/FM (C) [47], 25.9–29.9 µg/L [44], 41 µg/kg [43], 58 µg/L [49], 80 µg/L [45], 130 µg/L [41], 670 µg/L [46]
Apricot	1.52 µg/L [42]	0.46–0.78 µg/L [42], 6–9.2 µg/kg [50]	-	3.36–5.36 µg/L [42], 121 µg/L [49]
Blackcurrant	-	6 µg/kg/FM (C) [47], 8 µg/kg/FM (O), 17 µg/L [41]	-	0.011 µg/kg/FM (C) [47], 17 µg/kg/FM (O) [47], 110 µg/L [41]
Grapefruit	-	9 µg/L [41], 41 µg/L [46]	-	109 µg/L [41], 228 µg/L [46]
Kiwi	<LOD [49]	<LOD [42]	-	1.64 µg/L [42]
Orange	<LOD—3.02 µg/L [42], 0.7–0.9 µg/L [44], 65 µg/kg [43]	<LOD-20 µg/L [51], < LOD-0.64 µg/L [42], 0.6–0.7 µg/L [44], 6.4–9.2 µg/kg [50], 10 µg/kg [43], 10 µg/L [46], 10 µg/L [41]	0.8–0.9 µg/L [43]	1.02–10.03 µg/L [42], 15.9–16.1 µg/L [43], 80 µg/L [45], 91 µg/L [46], 95 µg/L [41]
Peach	<LOD—3.78 [42], 1.2 µg/L [44]	0.52–1.38 µg/L [42], 0.7 µg/L [44], 6.4–11.3 µg/kg [50]	0.8 µg/L [44]	1.94–18.58 µg/L [42], 30.7 µg/L, [44], 135 µg/L [49]
Pear	<LOD [42]	<LOD [42,46], 7 µg/kg/(C,O) [47]	-	1.62 µg/kg/FM [42], 10 µg/kg/FM (C, O) [47], 189 µg/L [46]
Pineapple	1 µg/L [44], 2.84 µg/L [42]	0.64 µg/L [42], 0.7 µg/L [44], 12 µg/L [46]	1.2 µg/L [44]	1.54 µg/L [42], 31.8 µg/L [44], 236 µg/L [46]

C—conventional, DM—dry mass, FM—fresh mass, LOD—limit of detection, O—organic.

**Table 7:** Values of some health risk indicators estimated by other authors in various products.

Type of Health Risk Indicator	Studied Element	Product/Group of Products	Results	References
HQ	Cd	Aronia juices	0.06–0.2	[1]
	Pb		0.03–0.2	
HI	Cd, Pb		0.072–0.491	
PTWI	Cd	Dried aronia berries	7 µg/kg/BW	[24]
	Pb		25 µg/kg/BW	
EDI	As	Tomato	6.0 × 10^−4^	[52]
		Cabbage	1.80 × 10^−3^	
	Cd	Tomato	1.76 × 10^−4^	
		Cabbage	4.90 × 10^−4^	
	Hg	Tomato	1.08 × 10^−3^	
		Cabbage	1.33 × 10^−3^	
THQ	As	Tomato	2.019	
		Cabbage	5.994	
	Cd	Tomato	0.176	
		Cabbage	0.490	
	Hg	Tomato	3.588	
		Cabbage	4.425	
HI	Al, Ba, Cr, Cu, Fe, Mn, Mo, Ni, Pb, Zn, Cr, Cu, Fe, Mn, Mo	Appple juice	6.35 × 10^−2^	[49]
		Pomegranate juice	6.26 × 10^−2^	
		Grape juice	4.170 × 10^−2^	
EDI	As	Apple juice	0.0347	[43]
		Orange juice	0.0353	
		Blackcurrant nectars	0.0429	
	Cd	Apple juice	0.0060	
		Orange juice	0.0054	
		Blackcurrant nectars	0.0060	
	Pb	Apple juice	0.0223	
		Blackcurrant nectars	0.0228	
		Orange juice	0.0060	
THQ	As	Apple juice	0.1158	[49]
		Orange juice	0.1176	
		Blackcurrant nectars	0.1430	
	Cd	Apple juice	0.1194	
		Orange juice	0.1086	
		Blackcurrant nectars	0.1194	
	Pb	Apple juice	0.0026	
		Orange juice	0.0022	
		Blackcurrant nectars	0.0027	
	Pb	Apple juice	5.370 × 10^−2^	
		Pomegranate juice	5.092 × 10^−2^	
		Grape juice	2.96 × 10^−2^	
	Pb	Different commercial juices (aloe vera, apple, cocoonut, lemon coconut, mango, mojito, multi-fruit, orange, peach, pineapple, pomegranate, strawberry, sour cherry)	6.26 × 10^−3^	[44]
	Hg		5.74 × 10^−3^	
	Cd		4.49 × 10^−3^	
	As		3.78 × 10^−3^	

## Data Availability

Data is contained within the article.
